# Linear and circular *PVT1* in hematological malignancies and immune response: two faces of the same coin

**DOI:** 10.1186/s12943-020-01187-5

**Published:** 2020-03-30

**Authors:** Martina Ghetti, Ivan Vannini, Clelia Tiziana Storlazzi, Giovanni Martinelli, Giorgia Simonetti

**Affiliations:** 1 Biosciences Laboratory, Istituto Scientifico Romagnolo per lo Studio e la Cura dei Tumori (IRST) IRCCS, Meldola, FC Italy; 2 Department of Biology, University of Bari Aldo Moro, Bari, Italy

**Keywords:** Non coding RNAs, PVT1, Hematological malignancies, Immune response

## Abstract

Non coding RNAs (ncRNAs) have emerged as regulators of human carcinogenesis by affecting the expression of key tumor suppressor genes and oncogenes. They are divided into short and long ncRNAs, according to their length. Circular RNAs (circRNAs) are included in the second group and were recently discovered as being originated by back-splicing, joining either single or multiple exons, or exons with retained introns. The human *Plasmacytoma Variant Translocation 1* (*PVT1*) gene maps on the long arm of chromosome 8 (8q24) and encodes for 52 ncRNAs variants, including 26 linear and 26 circular isoforms, and 6 microRNAs. *PVT1* genomic locus is 54 Kb downstream to *MYC* and several interactions have been described among these two genes, including a feedback regulatory mechanism. *MYC*-independent functions of *PVT1*/*circPVT1* have been also reported, especially in the regulation of immune responses. We here review and discuss the role of both *PVT1* and *circPVT1* in the hematopoietic system. No information is currently available concerning their transforming ability in hematopoietic cells. However, present literature supports their cooperation with a more aggressive and/or undifferentiated cell phenotype, thus contributing to cancer progression. *PVT1*/*circPVT1* upregulation through genomic amplification or rearrangements and/or increased transcription, provides a proliferative advantage to malignant cells in acute myeloid leukemia, acute promyelocytic leukemia, Burkitt lymphoma, multiple myeloma (linear *PVT1*) and acute lymphoblastic leukemia (*circPVT1*). In addition, *PVT1* and *circPVT1* regulate immune responses: the overexpression of the linear form in myeloid derived suppressor cells induced immune tolerance in preclinical tumor models and *circPVT1* showed immunosuppressive properties in myeloid and lymphoid cell subsets. Overall, these recent data on *PVT1* and *circPVT1* functions in hematological malignancies and immune responses reflect two faces of the same coin: involvement in cancer progression by promoting a more aggressive phenotype of malignant cells and negative regulation of the immune system as a novel potential therapy-resistance mechanism.

## Background

Non-coding RNAs (ncRNAs) are transcripts that do not encode proteins. They are diffused in the human genome and dysregulated in cancer cells. Given that genes for ncRNAs are often located in fragile sites (FRA), in regions with loss of heterozygosity and common breakpoint sites, they represent a new class of transcripts that participates in tumorigenesis [1, 2]. Some ncRNAs have a tumor suppressor function while others act as oncogenes [3, 4]. ncRNAs are classified into two categories on the basis of the length of their sequence: short ncRNAs do not exceed 200 nucleotides in size, while long ncRNAs (lncRNAs) are characterized by longer sequences. Short ncRNAs have been extensively studied. However, lncRNAs remain largely explored.

LncRNAs generally have a 5′ terminal methylguanosine cap, are frequently polyadenylated and alternatively spliced [5, 6]. They have a thermodynamically stable secondary structure with hairpin loops and bulges [7], that enables them to interact with DNA, mRNAs, ncRNAs and proteins. lncRNAs regulate gene expression at different levels, from mRNA translation to cytoplasmatic and nuclear epigenetic processes, including miRNA sponging [8].

Circular RNAs (circRNAs) represent an emerging group of cellular lncRNAs. They are covalently closed loop-like structure with no 5′ and 3′ polarity and this circular structure confers them an increased stability and resistance to the cellular linear RNA decay machineries [9]. Evidence suggests that circRNAs have an independent biogenesis, which is unrelated to canonical splicing of linear RNA. Indeed they result from a back-splicing of the 5′ splice position with the 3′ splice position, or from exon skipping [10]. circRNAs expression is partially regulated by DNA methylation of host genes. In particular, a recent study showed that knockdown of DNMT3A induces circRNAs expression in a host gene dependent manner, while changes in their level is largely independent of host gene regulation upon silencing of DNMT3B [11]. Once synthesized, circRNAs tend to form 16–26 base pair intra-molecularly imperfect RNA duplexes and can be degraded by RNase L upon activation of early innate immune response [12].

**Fig. 1:**
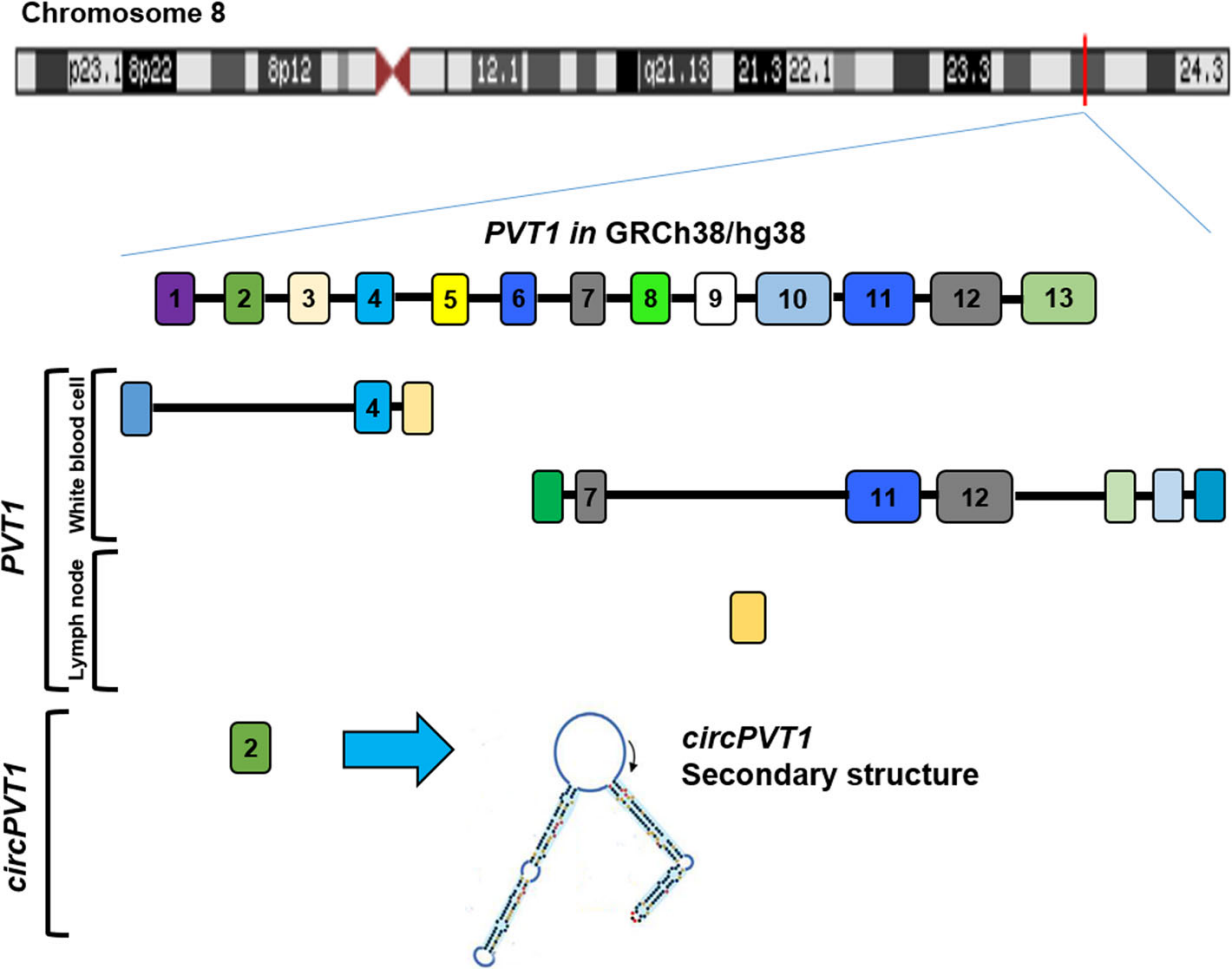
*PVT1* transcript alignment in UCSC Genome Browser on Human Dec. 2013 (GRCh38/hg38) Assembly. 8q24.21 region encodes for *PVT1* and *circPVT1.* The sequence of *PVT1* transcript with the largest number of exons is shown in the upper part of the figure. *PVT1* isoforms and *circPVT1* with the highest expression in the hematopoietic system, are shown in the lower part of the figure (from the top: NONHSAT255418.1, NONHSAT255436.1, NONHSAT255445.1). The secondary structure of *circPVT1* is reported as discovered by Liu et al. [12]. The rectangles represent the exons

Human *Plasmacytoma Variant Translocation 1* (*PVT1*) gene is located on chromosome band 8q24.21 (Fig. 1) and encodes for both circRNAs and linear ncRNA isoforms, as well as 6 microRNAs. In this review, we describe the currently available data on the role of both *PVT1* and *circPVT1* in hematopoietic cells and especially in hematological malignancies, including genomic alterations, involvement in disease progression and in the regulation of the immune response, which represents a potential therapy-resistance mechanism.

### *PVT1* and *circPVT1*: different isoforms with a different regulation

**Fig. 2:**
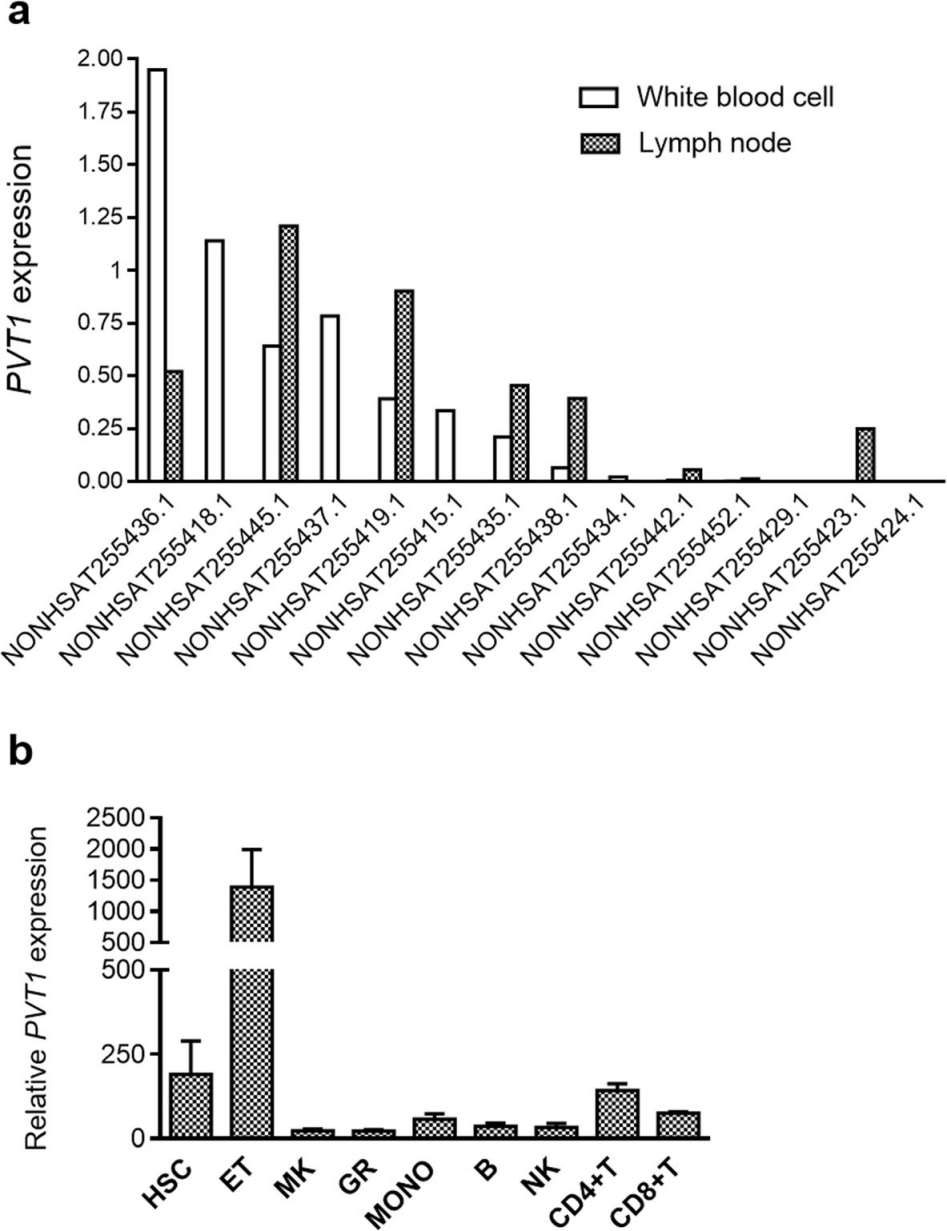
*PVT1* expression in the hematopoietic system. **a***PVT1* isoforms detected in lymph node and/or white blood cells (www.noncode.org). FPKM from Illumina’s Human BodyMap 2.0 project are shown (http://www.ensembl.info/2011/05/24/human-bodymap-2-0-data-from-illumina/). **b** Overall *PVT1* expression in hematopoietic cell populations (GSE98791). Data from Agilent-021441 NCode Human Long Non-coding RNA microarray were analyzed with Feature Extraction Software10.5 (Agilent) [18]. The processed signal intensity of *PVT1* is represented in the figure (HSC: hematopoietic stem cells, ET: in vitro-differentiated erythroblasts, MK: in vitro-derived megakaryocytes, GR: granulocytes, MONO: monocytes, B: B lymphocytes, NK: natural killer cells, CD4 + T: CD4^+^ T lymphocytes, CD8 + T: CD8^+^ T lymphocytes)

Twenty-six *circPVT1* isoforms have been annotated in the CircInteractome Database (https://circinteractome.nia.nih.gov/index.html) [13], with a spliced length ranging from 113 to 11,130; 8 of them were also detected in the K562 and Gm12878 hematopoietic models, among others. The most common isoform is 410 bp. It is a product of back-splicing and contains the whole exon 2 of *PVT1*, forming a closed loop-like structure [14, 15]. Conversely, *PVT1* exists in 26 different transcript variants (www.noncode.org) [16], with some of them not containing exon 2. The different variants are capped at the 5′ and polyadenylated at 3′ end [17]. *PVT1* isoforms are variably expressed across human tissues, with adrenal gland and heart displaying the highest expression (www.noncode.org). Hematopoietic tissues, namely lymph node and white blood cells, showed high levels of few isoforms (Fig. 1), while most of them were barely detectable (Fig. 2a).

*PVT1* and *circPVT1* are transcribed from two different promoters, thus confirming an independent regulation of their expression, with *circPVT1* promoter being upstream of exon 2 [19]. Accordingly, Chen et al. reported that the expression levels of *PVT1* and *circPVT1* were poorly correlated in gastric cancer tissue and human gastric epithelium GES-1 line [20]. Moreover, an independent post-transcriptional regulation of *PVT1* and *circPVT1* has been suggested on the basis of their structure and localization. *circPVT1* mostly localizes in the cytoplasm, whil*e PVT1* is primarily nuclear [21]. In addition, thanks to its circular structure, *circPVT1* is resistant to exonuclease activity of RNase R and it was suggested to form a protein-coding open reading frame (ORF) of 104 amino acids [22], whose expression and role across cancer deserves further investigation. Regarding their function, both isoforms are upregulated in many cancer types and correlated with various clinical features, including overall survival and lymph node metastases [23]. However, these associations may vary among cancer types and the specific *PVT1* isoform (linear or circular), clearly suggesting the need to disentangle their biological function at cellular level [24].

The majority of studies focuses on the linear isoform of *PVT1*, that promotes cell growth and proliferation in cancer, as well as cell migration, invasion, and drug resistance. Moreover, a role as a sponge for tumor-suppressor miRNAs with oncogenic properties has been described for both *PVT1* isoforms, even though the literature points on different miRNA entities, depending on cancer type, especially for *circPVT1*. Their pro-tumorigenic role is often attributed to their functional interaction with the *MYC* oncogene, localized about 54 Kb upstream *PVT1*. However, recent findings provided new insights on potential MYC-independent functions, especially for *circPVT1*, which will be addressed in the following sections.

### Role of *PVT1* and *circPVT1* in the immune system

LncRNAs and circRNAs have been reported to participate in the differentiation and functioning of immune cells under physiological [25] and pathological conditions [26]. In the hematopoietic lineage, *PVT1* is expressed in CD34^+^CD38^−^ cord blood-derived stem cells [18] (GSE98791, Fig. 2b). Moreover, in mature cells, high levels of this transcript were detected in peripheral blood (PB) T lymphocytes (CD4^+^ > CD8^+^) and, mostly, in vitro-differentiated erythroblasts (Fig. 2b). Accordingly, Gillinder et al. reported an increase in *PVT1* transcription early after erythropoietin (EPO) stimulation in the murine immature erythroid J2E cell line [27], that proliferates and terminally differentiates following EPO exposure. However, the role of *PVT1* in the erythroid lineage has not been further elucidated.

*PVT1* expression, including extracellular and intracellular RNA, increased in the PB of mice 16, 24 and 48 h after whole body irradiation (2–8 Gy) [28], along with other p53-related genes. Since radiation leads to DNA damage, with consequent activation of the p53-dependent DNA repair pathways and since p53 binding to its responsive element contributes to *PVT1* upregulation [29], *PVT1* may serve as a biomarker of DNA damage response. Therefore, *PVT1* might be potentially translated to the clinics for an early evaluation of ablative regimens or as an easy-to-use readout of activation of a DNA damage response.

In parallel, preclinical and clinical data suggest a potential role for *PVT1* and/or *circPVT1* in the myeloid and lymphoid lineages. From a structural point of view, a genome wide association study identified 44 variants in the *PVT1* gene correlating with selective IgA deficiency [30]. The peak *PVT1* variant was in moderate linkage disequilibrium with four variants with a potential regulatory role (rs1499364, rs7001706, rs35135218, rs10601187) that were predicted to affect binding and were located in transcription factors binding sites. In particular, rs1499364 lies in a region of open chromatin and in a histone mark for active transcription in regulatory T (Treg) cells. Moreover, the intronic variant rs7001706 is located in a H3K4me1 histone mark in Treg cells and in a FOXP3 transcription factor binding motif. Of note, IgA deficiency, which results in defective regulation of mucosal immunity and gut commensalism, with recurrent mucosal infections [31, 32], shows a higher penetrance in families with autoimmunity recurrence. In particular, the prevalence of systemic lupus erythematosus (SLE), type 1 diabetes and celiac disease are respectively 10 and 35 times higher in patients affected by IgA deficiency, compared with the general population [33].

At functional level, *circPVT1* is significantly reduced in monocytes, B and T lymphocytes from the PB of SLE patients, along with other circRNAs having intra-double stranded (ds) RNA duplexes, while the expression of their linear cognate mRNAs is marginally affected [12]. The reduced level of circRNAs in SLE patients has been linked to the spontaneous activation of RNase L [12], a cytoplasmic endoribonuclease that is generally activated by pathogenic dsRNAs or viral infection [34]. RNase L-mediated degradation of circRNAs, including *circPVT1*, has been also demonstrated in acute T cell leukemia and acute monocytic leukemia cellular model and is responsible for hyperactivation of the interferon (IFN)-inducible serine/threonine protein kinase PKR, that physiologically occurs in the early stage of the innate immune responses [12]. Exogenous expression of circRNAs reduced PKR activation and EIF2α phosphorylation in T cells from SLE patients and suppressed IFN-β and type I IFN-induced gene signatures, which are hallmarks of SLE. These data indicate a role of *circPVT1* in the regulation of immune responses and the possibility of using circRNAs as potential therapies against autoimmune diseases. In addition, reduced expression of *PVT1* was reported in PB cells of relapsing-remitting multiple sclerosis patients compared with healthy subjects [35] and a role for *PVT1* has been reported in the inflammatory processes involved in asthma [36] and septic acute kidney injury [37].

While evidence is available through the literature regarding the immunoregulatory role of *PVT1* and/or *circPVT1* under infection, inflammatory conditions and autoimmune disease, little is known about their function in the immune system during malignant transformation. High expression of linear *PVT1* has been detected in granulocytic myeloid-derived suppressor cells (G-MDSC) from tumor tissues in murine models of Lewis lung carcinoma and colorectal cancer [38]. *PVT1* expression was induced by HIF-1α in tumor-infiltrating G-MDSC, which experiment hypoxic conditions. In these cells, *PVT1* regulates ARG1 activity and reactive oxygen species (ROS) production, thus contributing to the suppression of T-cell-induced antitumor immune responses. Indeed, the proportion of CD4^+^ IFN-γ^+^ T helper 1 and CD8^+^ IFN-γ^+^ cytotoxic T lymphocytes was increased in tumor tissues (the latter also in the draining lymph nodes) of mice injected with G-MDSC lacking *PVT1* expression.

These data on the MDSC-mediated function of *PVT1* suggest that its overexpression in specific immune cell subsets has the capability of dampening anti-tumor responses and potentially contribute to therapy resistance, especially in the therapeutic settings relying on immune cell reactivation (e.g. immune checkpoints-based regimens, which are largely exploited for combination strategies). Moreover, the observation that *PVT1* is highly expressed in the T cell lineage and that *circPVT1* levels are reduced in lymphocytes from SLE patients suggest novel potential direct implications of either the linear and the circular isoform in the T cell-mediated anti-tumor response.

### *PVT1* and *circPVT1* in hematological malignancies

Chromosome 8q24.21 is a target for genomic rearrangement across cancer, including hematological malignancies. Particularly, it is frequently involved in the emergence of aberrant chimeric genes, high copy number gains, both of them associated with poor prognosis in human cancers. Moreover, it harbors a number of susceptibility loci at 8q24.21 near or in the *PVT1* gene, such as single nucleotide variants in different types of lymphoma (Table 1).

**Table 1:** *PVT1* and *circPVT1* structural and functional alterations in hematological malignancies

Hematological malignancy	***PVT1***	Molecular alteration	Downstream genes (direct or indirect regulation)	Functional role	References
AML	Linear	Genomic amplification, rearrangements (*PVT1-CCDC26* *PVT1-NSMCE2*), upregulation	MYC	↑ proliferation, ↓ apoptosis, maintenance of an undifferentiated phenotype	[39–44]
AML	Circular	Genomic amplification, rearrangements			[41]
APL	Linear	Genomic amplification, upregulation	MYC	Maintenance of an undifferentiated phenotype, cell cycle progression	[45–47]
AEL	Linear	Upregulation	MYC, p15, p16, BCL2	↑ proliferation, ↓ apoptosis and necrosis	[17, 48, 49]
B-ALL	Circular	Upregulation	MYC, BCL2	↑ proliferation, ↓ apoptosis	[50, 51]
T-ALL	Circular	Upregulation	MYC, BCL2	↑ proliferation, ↓ apoptosis	[50]
T-ALL	Linear	Upregulation	MYC, p15, p16, BCL2, Caspase-3	↑ proliferation, ↓ apoptosis	[52]
CLL	Linear	t(8;13) (q24;q14) and deletion, upregulation			[53]
BL	Linear	t(2;8), t(8;22)	MYC, CDKN2A, CDN1B, RB1, CCND2, GADD45A, CDC20, CDK4, CD6, ATM, BRCA2	↑ proliferation	[54–58]
HL	Susceptibility loci at 8q24.21 near or in the *PVT1* gene	rs2019960, rs2608053			[59, 60]
DLBCL	Susceptibility loci at 8q24.21 in close proximity to *PVT1,* focal promoter deletions, amplification	rs13255292, and rs4733601	MYC, BCL2	double-hit-like expression pattern (focal deletions of promoter)	[61–64]
FL	Susceptibility locus at 8q24.21 near *PVT1*	rs13254990			[65]
MM	Linear	Genomic amplification, translocations, upregulation	MYC, BCL2, miR-203	↑ proliferation, ↓ apoptosis	[48, 66–69]
MM	Circular	Upregulation	BCL2, Caspase-3, PARP	↑ proliferation, ↓ apoptosis, resistance to glucocorticoid treatment	[70]

### Acute myeloid leukemia

Chromosomal rearrangements and copy number changes at the 8q24.21 locus are relatively frequent events in acute myeloid leukemia (AML) and play a role in its pathogenesis. Retroviral insertion analysis from various non-T cell derived mouse tumors identified the first integration at the *pvt1* locus in myelogenous leukemia [71]. The first evidence of *PVT1* involvement in human AML came from cytogenetic studies on the 8q24 locus and on double minutes chromosomes (dmin) [39]. Indeed, a 4.3 Mb minimal common amplicon was identified in *MYC*-containing dmin, with clustered distal breakpoints located downstream the *PVT1* gene, among others [40]. A t(6;8)(p21;q24) translocation involving the TATA-binding protein-associated factor *SUPT3H* and *PVT1* gene has been recently reported in blastic plasmacytoid dendritic cell neoplasm, with breakpoint regions mapping in exon 3 and exon 1, respectively [72]. In AML, *PVT1* appeared as a breakpoint hotspot in *MYC* amplification. Indeed, 92% of AML cases carrying *MYC* amplifications as dmin, homogeneously staining region (hsr), or ring chromosomes (AML-amp) were characterized by expression of chimeric transcripts that frequently involved *PVT1* as either a 5′ or 3′ partner, with *PVT1* amplification. *PVT1* fusion genes were generated as post-transcriptional events, since they were not identified at genomic level and showed a conserved breakpoint position (in the majority of cases) and *MYC*, *FAM49B*, *RP11-89 K10*, *CCDC26*, *CASC11*, and *CASC8* as recurrent partners, with a predicted dysregulation of their protein product due to promoter swapping, loss of the untranslated region or N/C-terminus truncation, in case of protein coding genes as partners in fusions. For chimeras joining *PVT1* with other non-coding transcripts, the role is presently unclear. The *PVT1-CCDC26* and *PVT1-NSMCE2* chimeras were also detected in AML cell lines [41] and primary cells [42], respectively, and *PVT1*-*NSMCE2-*rearranged AML showed amplification of both genes and relocation in micronuclei [42]. *PVT1* and *NSMCE2* overexpression and involvement in chimeric transcripts were also specific features of AML-amp cases with the highest numbers of chimeras. AML-amp cases carrying *PVT1* amplification showed increased levels of *PVT1* and *circPVT1*, the latter being also confirmed in AML cell lines with more than 5 copies of *PVT1* [41]*.*

**Fig. 3:**
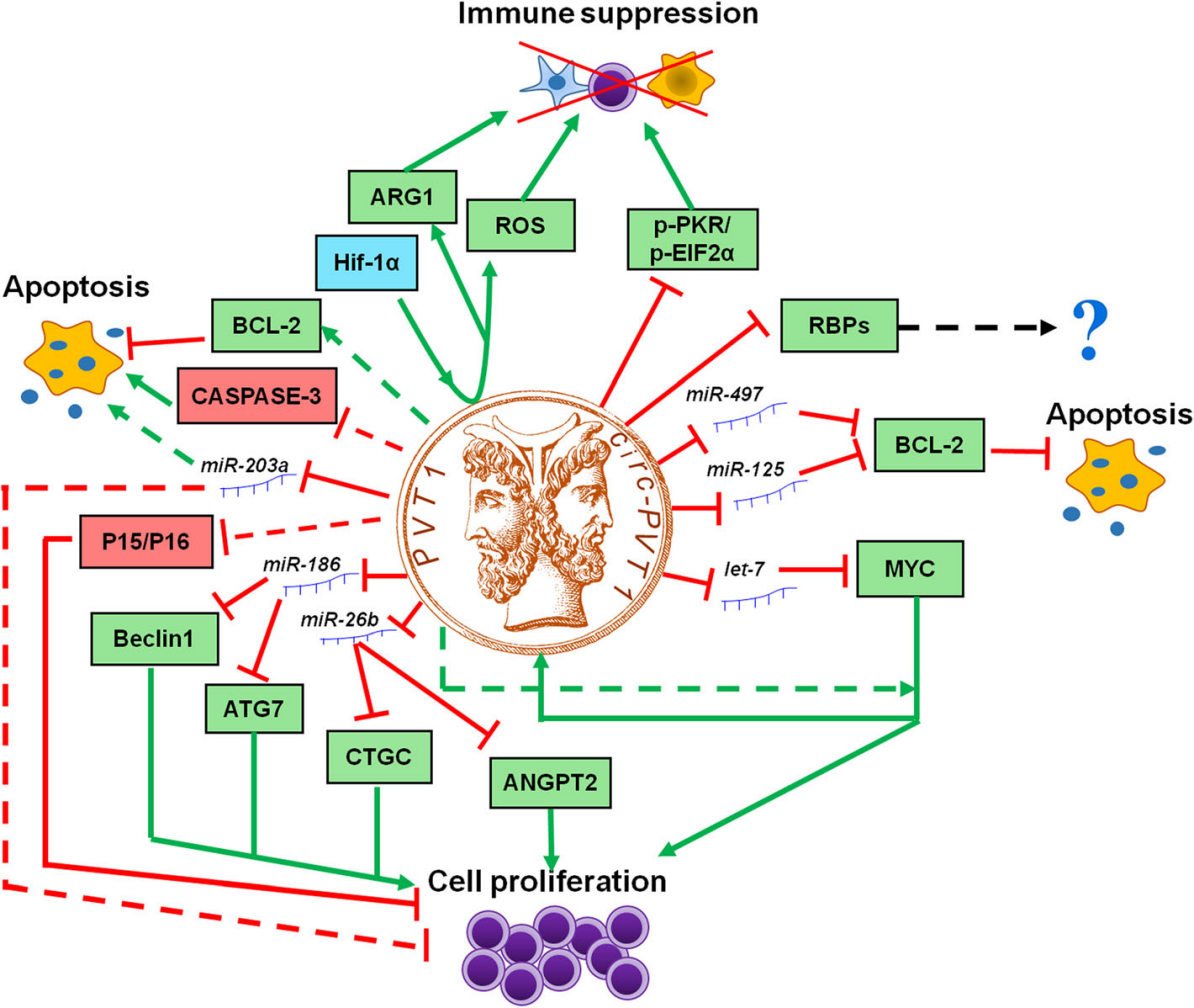
Model of *PVT1* and *circPVT1* pro-tumorigentic functions in hematological malignancies. *PVT1* and *circ-RNA* act as a miRNA sponge and regulate the energy metabolism, protein stability, cell cycle progression through a variety of pathways, thus promoting cell proliferation, immune suppression and inhibiting cell death (green represents induction, red indicates suppression, dashed line shows indirect and not specified mechanisms; ROS: reactive oxygen species; RBPs: RNA-binding proteins, including EIF4A3, U2AF65, AGO2, AUF1, DGCR8, FUS, HNRNPC, PTB, TAF15, TDP43, TIA1, TIAL1, LIN28A)

Conversely, in the general AML population, controversial results have been reported regarding *PVT1* expression levels in bone marrow (BM) blasts compared with healthy donors [43, 45, 73]. However, different groups agreed on the increased *PVT1* level in acute promyelocytic leukemia (APL), compared with mononuclear cells [45] or granulocytes [46] from healthy donors. Genomic amplification of the 8q24 chromosomal region is a common secondary event in human APL [47], indicating that *PVT1* may be involved in APL progression. *PVT1* expression was downregulated, along with MYC, by all-trans retinoic acid (ATRA) in APL models, thus indicating a potential role for the lncRNA in ATRA-induced granulocytic differentiation [74] and cell cycle arrest [46]. MYC silencing was sufficient per se to reduce *PVT1* levels. In turn, *PVT1* knockdown resulted in decreased MYC protein, with no changes at mRNA level, clearly showing a dual relationship between *PVT1* and *MYC*, the former controlling MYC protein synthesis and/or stability in malignant promyelocytes. This evidence suggests that *PVT1* contributes to an aggressive phenotype in APL, by modulating cell proliferation. What comes first, whether MYC or *PVT1*, remains unresolved. The role of *PVT1* in APL progression is also supported by its upregulation in high risk APL (white blood cell count> 10,000/mL) compared with intermediate and low risk cases (defined according to white blood cell and platelet count) [45]. A prognostic or functional role of *PVT1* has also been reported in other AML subtypes. AML harbouring the t (8;21) translocation have higher *PVT1* compared with other AML. In particular, high *PVT1*, or high *MYC* predicted shorter leukemia-free survival in t(8;21) AML patients [43], which suggested a potential mechanism of chemotherapy resistance in cells with leukemia initiating capacity, that drives disease progression. This hypothesis would reinforce the observation that *PVT1* knockdown reverses multidrug resistance in solid tumors [75]. In line with its elevated level in normal erythroid cells, *PVT1* was highly expressed in acute erythroleukemia (AEL) models [48]. Its inhibition led to a significant decrease of cell proliferation, with accumulation of cells in the G0/G1 phase of the cell cycle, that associated with downregulation of MYC protein and upregulation of p15 and p16 [48] (Fig. 3). BCL2 was also targeted by *PVT1* silencing, thus resulting in induction of apoptosis and necrosis [17, 48, 49]. Consistently with the results obtained in APL, in the murine MLL-AF9/NRAS^G12D^ and MLL-ENL AML models, *PVT1* depletion activated a myeloid differentiation program, with downregulation of cKit and leukemia stem cell signatures and upregulation of CD11b [44]. This phenotype resembled the one induced by bromodomain and extra-terminal domain (BET) protein inhibitors in AML [76] and was mediated by MYC downregulation, since its ectopic expression reversed the differentiation and anti-proliferative programs promoted by *PVT1* silencing [44].

### Acute lymphoblastic leukemia

Acute lymphoblastic leukemia (ALL) is the only hematological malignancy in which a functional role of *circPVT1* has been clearly demonstrated. Indeed, *circPVT1* (but not *PVT1*) was specifically overexpressed in primary BM cells from B- and T-ALL, compared with healthy controls [50]. Notably, higher levels of the transcript were detected in younger patients (aged less than 35 years). Moreover, higher *circPVT1* levels were detected in pediatric B-precursor ALL patient-derived xenograft samples, and in particular in *ETV6-RUNX1* rearranged cases, compared with B cells from healthy donors [51]. Downregulation of *circPVT1* in B- and T-ALL models had no effect on *PVT1* (as also observed in gastric cancer) [20], while causing a significant reduction in the proliferation rate and induction of apoptosis, associated with a decrease of MYC and BCL2 protein expression [50] (Fig. 3). The observations that *circPVT1* primarily localizes in the cytosol strongly support a role as miRNA sponge [77], reinforcing the hypothesis of a post-transcriptional regulation of *circPVT1* over its target genes [50]. In particular, *circPVT1* seemed to sponge *let-7* and *mir-125*, that are known to modulate *MYC* and *BCL-2*, respectively [52] (Fig. 3). In parallel, *PVT1* knockdown in the Jurkat T-ALL cell line also suppressed cell proliferation, with cell cycle arrest in G0/G1 phase, increased apoptosis [52] and upregulation of Caspase-3, p15, and p16 expression (Fig. 3). BCL2 and MYC were downregulated by *PVT1* knockdown in T-ALL cells, as observed upon *circPVT1* silencing [50] in B- and T-ALL (Fig. 3). These results may suggest a similar, but not compensatory, function of *PVT1* and *circPVT1* in ALL. However, we cannot exclude that downregulation of *circPVT1*, potentially occurring during *PVT1* silencing, might be the driver of the observed phenotype. Specific experimental and cell engineering strategies will be required to clarify this question.

### Chronic lymphocytic leukemia

Differently from other hematological malignancies, 8q24 rearrangements are generally rare (3.7% of amplified cases [78]) in B-cell chronic lymphocytic leukemia (CLL) and they are often acquired during the disease course [79]. Gain of 8q24 associates with poor overall survival and/or shorter time to first treatment [78, 80, 81] and is frequently detected in 17p-deleted CLL, where it has a negative prognostic value [82]. *PVT1* has been investigated in a single CLL case with complex karyotype, t(8;13)(q24;q14) translocation and a deletion on the derivative chromosome 8 mapping downstream the *MYC* oncogene and encompassing the *PVT1* locus [53]. Nevertheless, *PVT1* was significantly upregulated, as well as *MYC*, as a consequence of the t(8;13) translocation and might have a potential role in the aggressive phenotype of that CLL case.

### Lymphomas

The first *PVT1* alterations identified in lymphoma refer to Burkitt lymphoma (BL) cases carrying the t(2;8) or t(8;22) translocations (~ 20%), that juxtapose the *IGL* or *IGΚ* locus with the *PVT1* gene, resulting in chimeric transcripts that contain *PVT1* exons 1a or 1b spliced to the *IG* light chain constant region [54–56]. *PVT1* was recently reported as a mutational hotspot in endemic and sporadic BL [57]. Its downregulation in the BL Raji model decreased MYC protein expression and suppressed proliferation by promoting cell cycle arrest in the G0/G1 phase [58]. Accordingly, cell cycle-and DNA damage response-related genes were altered by *PVT1* knockdown, including upregulation of *CDKN2A*, *CDN1B*, *RB1*, *CCND2*, *GADD45A* and downregulation of *CDC20*, *CDK4*, *CD6*, *ATM* and *BRCA2*.

Genomic events targeting the *PVT1* locus have been also described in other lymphoma types. *Pvt1* is the third most frequent murine leukemia virus (MLV) integration site, driving T cell lymphomas in mice [83, 84] and rats [85, 86]. Tumors induced by retroviral insertions into the locus, that mainly clusterized around exon 1, where characterized by overexpression of the exon 1 of *pvt1* and of its microRNA product, mmu-miR-1204, which is encoded by exon 1 as well. Moreover, MLV integration in the *pvt1* locus variably co-occurred with insertions tagging *evi5*, *notch1*, *rasgrp1*, *ahi1*, *gfi1*, but not *myc* [84]. The mutual exclusivity between *pvt1* and *myc* genomic rearrangements reinforces the hypothesis of their reciprocal relationship in terms of expression and biological function.

A number of genome-wide association studies on Hodgkin’s lymphoma (HL), diffuse large B cell lymphoma (DLBCL) and follicular lymphoma (FL) identified novel susceptibility loci at 8q24.21 near or in the *PVT1* gene. In particular, rs2019960, encompassing intron 6 of *PVT1* and rs2608053, localized telomerically to *PVT1,* were associated with classical HL risk, with poor correlation between each other [59]. The rs2608053 susceptibility locus (GG vs AG + AA) was also predictive of patients’ outcome in terms of progression free and overall survival in HL [60]. Two independent variants, rs13255292 and rs4733601, located telomerically to the 8q24 region, in close proximity to *PVT1,* were recognized as risk factors for DLBCL [61] and one of them (rs13255292) was confirmed in the Eastern Asian population [62]. Moreover, a new susceptibility locus (rs13254990) mapping near *PVT1*, was associated with FL risk [65]. In DLBCL, focal deletions of the *PVT1* promoter was suggested to promote *MYC* overexpression and a double-hit-like expression pattern in germinal center type tumors lacking *MYC* and/or *BCL2* rearrangements [63].

Despite the frequency of genomic alterations and rearrangements, few studies investigated the functional role of *PVT1* in lymphomas. Recently, a novel cell line, named AMU-ML2, characterized by the occurrence of a homogeneously staining region at the 8q24 locus and containing more than 20 copies of the entire *MYC* and *PVT1* genes, has been established from a DLBCL patient at diagnosis. Of note, AMU-ML2 cells expressed elevated levels of *MYC*, *PVT1* and *circPVT1* and were resistant to vincristine, suggesting a potential link between *PVT1* and drug resistance in DLBCL [64]. This hypothesis is in line with previous reports on *PVT1*-mediated resistance to cisplatin in gastric and ovarian cancers [87].

### Multiple myeloma

*PVT1* is frequently involved in translocations occurring in multiple myeloma (MM) and murine plasmacytoma, where the t(6;15) (*igκ*-*pvt1*) and the t(15;16) (*pvt1*–*igλ*) fusion genes [88, 89] have been reported to upregulate *PVT1* expression [90]. Various partners loci were described in MM with 8q24 abnormalities, including 4p16, 4q13, 13q13, 14q32, and 16q23–24 [66]. In particular, the *PVT1-NBEA* and *PVT1-WWOX* chimeras were highly expressed in the AMU-MM1 and RPMI8226 cell lines harboring t(8;13)(q24;q13) and der(16)t(16;22)ins(16;8)(q23;q24) rearrangements, respectively [66]. Moreover, co-amplification of *MYC* and *PVT1* has been also reported in MM [67].

Elevated *PVT1* levels were detected in MM BM cells compared with normal tissue [68] and primarily in *MYC*-rearranged MM cases [69]. In particular, high *PVT1* was associated with MM carrying recurrent *MYC* fusion genes (with *IGH*, *IGK*, *IGL*, *TXNDC5*/*BMP6*, *FOXO3* and *FAM46C* partner genes) or complex rearrangements involving more than five loci, or hyperdiploid cases, that also overexpressed MYC [69]. Despite the weak correlation observed between *MYC* and *PVT1* expression in MM, a MYC binding site in the *PVT1* gene was experimentally validated in MM cells, thus suggesting a positive feedback loop between the two genes, sustaining their elevated expression [69, 91]. Taken together, this evidence could depict a novel molecular paradigm underlying the pathogenesis of 8q24 rearrangement-positive MM. *PVT1* knockdown in MM cell lines inhibited cell proliferation and promoted apoptosis [48] through restoration of expression of miR-203a [68] (Fig. 3). Indeed, *PVT1* acts as a miR-203a sponge and silencing of miR-203a reversed the *PVT1*-knockdown phenotype. A similar function has been recently suggested for *circPVT1*, whose ectopic expression enhanced the proliferation rate of MM models, suppressed apoptosis and expanded the stem cell compartment [70]. Moreover, recent findings point to *PVT1* and *circPVT1* role in treatment response and resistance. *PVT1* was downregulated by BET inhibitors, along with MYC [92]. However, it was not altered by inhibitors of MYC transcriptional activity, suggesting a BRD4-mediated co-regulation of the two genes, rather than a MYC-dependent expression of the lncRNA. Moreover, *circPVT1* is overexpressed in glucocorticoid resistant cells and its downregulation enhanced sensitivity to glucocorticoid treatment, induced apoptosis and inhibited cell proliferation in resistant cell lines and xenograft models through upregulation of Caspase-3 and PARP and downregulation of BCL2 [70].

### *PVT1* and *circPVT1*: MYC partners in crime and beyond

Due to their close proximity at the 8q24 locus, *PVT1* and *MYC* are often considered tween players, and a positive interaction feedback loop has been demonstrated in solid tumors [93] and in APL [46] and MM [91], among hematological malignancies. It is doubtless that their co-expression does not occur by chance. Indeed, normal tissues generally express *MYC* but very low levels of *PVT1*, while transformed cells also display elevated *PVT1* [91, 93]. Moreover, BRD4 has been suggested to regulate the expression of both *PVT1* and *MYC* in MM [92]. By comparing transcripts correlated with *PVT1* expression across myeloid and B cell malignancies (adult and pediatric AML, pediatric ALL and DLBCL, data available through the cBioPortal database, https://www.cbioportal.org) we identified a core of 169 common genes, showing a positive or negative correlation with *PVT1* (Spearman *q* ≤ 0.05, Additional file 1). Of note, 9 of them mapped at the 8q24 locus (*CYC1*; *TSTA3*; *SLC39A4*; *PUF60*; *SHARPIN*; *ADCK5*; *MFSD3*; *FBXL6*; *HSF1,* fold discovery rate [FDR] = 0.0002 from http://www.webgestalt.org/2017/option.org [94]), suggesting a positional effect.

The biological consequences of *PVT1* and *circPVT1* alterations are often attributed to the downstream deregulation of MYC. Evidence obtained across hematological malignancies indicates that the regulatory role exerted on cell proliferation by *PVT1* (in AML, T-ALL, BL, MM) and *circPVT1* (in T-ALL, B-ALL) is mediated by MYC, as also observed in a number of solid tumors [95, 96], and this regulation is active at post-transcriptional level (Fig. 3). Several studies have suggested that *PVT1* enhances the stability of MYC protein by preventing its phosphorylation at threonine 58 and subsequent degradation [95, 97]. Moreover, *PVT1* can also bind to *MYC* transcript and regulate its expression [97]. No correlation between *MYC* transcript and *PVT1* was reported in adult and pediatric AML, pediatric ALL and DLBCL, differently from observations obtained in pan-cancer cohorts, which is indicative of a lower frequency of genomic amplification occurring at the 8q24 locus [93]. Conversely, enrichment of a MYC-related signature was identified among *PVT1* coexpressed genes in hematological malignancies (*QTRT1*; *TSFM*; *ZNF593*; *NDUFAF4*; *CCDC124*; *MON1A*; *RRP9*; *ISOC2*, adjusted *p* = 0.04, MSigDB oncogenic signatures, https://amp.pharm.mssm.edu/Enrichr/enrich [98]), further reinforcing the post-transcriptional nature of the MYC-*PVT1* interplay.

Along with MYC-related genes, pathway analysis highlighted a significant enrichment of transcripts involved in transcription, RNA metabolism, translation, oxidative phosphorylation, purine and pyrimidine metabolism (Additional file 2), suggesting a potential role of *PVT1* in these cellular processes. The correlating genes may be either regulated by *PVT1* and/or co-regulated with the lncRNA itself. In addition, *PVT1* was reported to recruit EZH2 [99] to *LATS2* [100], *CDKN2B* and *CDKN2A* [101] promoters, in order to repress their transcription, suggesting a MYC-independent activity. Although this evidence was obtained in solid tumor models, the involved genes, and in particular the epigenetic regulator EZH2 are well known also in hematological malignancies [102], thus deserving further investigation.

The oncogenic role of *PVT1* and *circPVT1* has also been linked to their function as competing endogenous RNA (ceRNA) through binding of tumor suppressor microRNAs and *circPVT1* could serve as sponges for RNA-binding proteins (RBPs, Fig. 3). Indeed, binding sites for EIF4A3, U2AF65, AGO2, AUF1, DGCR8, FUS, HNRNPC, PTB, TAF15, TDP43, TIA1, TIAL1, LIN28A were predicted on *circPVT1*, with the first 3 RBPs showing the strongest evidence (https://circinteractome.nia.nih.gov/index.html). Among the microRNAs regulated by *PVT1*, miR-26b, miR-203a, miR-214, miR-424 and miR-497 were reported to be deregulated and play a role in the pathogenesis of lymphoma [103–108] and/or MM [68, 109–113] and/or leukemia [114–121]. mir-26b appeared among those showing a significant interaction with the core of *PVT1*-coexpressed transcripts (*p* ≤ 0.05, mirTarbase, https://amp.pharm.mssm.edu/Enrichr/enrich), along with miR-186 and miR-16, which are also sponged by *PVT1* and are involved in the regulation of cell cycle and apoptosis [122]. Of note, it was recently reported that *PVT1* also regulates cell migration and angiogenesis through miR-26b and miR-186, along with direct interaction with RBPs and/or signaling molecules. Indeed, *PVT1*-binding of miR-186 led to upregulation of the autophagy-promoting genes ATG7 and Beclin1, with increased migration of glioma vascular endothelial cells [123]. Similarly, *PVT1* enhanced in vitro vascular tube formation of HUVECs by enforcing the expression of CTGF and ANGPT2, through miR-26b suppression [124]. The pro-angiogenic function of *PVT1* is also exerted through direct interaction with phospho-STAT3, leading to protein stabilization and activation of the downstream pathway, resulting in *VEGFA* upregulation in gastric cancer [125]. Moreover, *PVT1* regulates VEGF in non-small-cell lung cancer and ANGPTL4 in cholangiocarcinoma by acting as miR-29c sponge [126] and by binding to the epigenetic modification complex PRC2 [127], respectively. These data, obtained in solid tumors, can offer new insights in hemato-oncology, and in particular in MM, where angiogenesis is a hallmark of disease progression [128].

Recent evidence on the role of *PVT1* in immune system opens a new scenario, in which the link with MYC has not been completely addressed. Indeed, MYC is a downstream target of *PVT1* in G-MDSC, but its involvement in the regulation of their immune suppressive function, including ROS production and ARG1 activity, has not been fully elucidated [38]. Of note, Zheng et al. proposed a microenvironmental regulation of *PVT1* expression, mediated by HIF-1α under hypoxic stress [38]. Similarly, levels of *circPVT1* and other circRNAs are physiologically regulated or pathologically deregulated by RNase L in immune cells, resulting in changes of PKR activity and, consequently in T cell functionality [12]. These data point to novel putative MYC-independent functions of *PVT1* and *circPVT1* in (anti-tumor) immune response.

## Conclusions

**Fig. 4:**
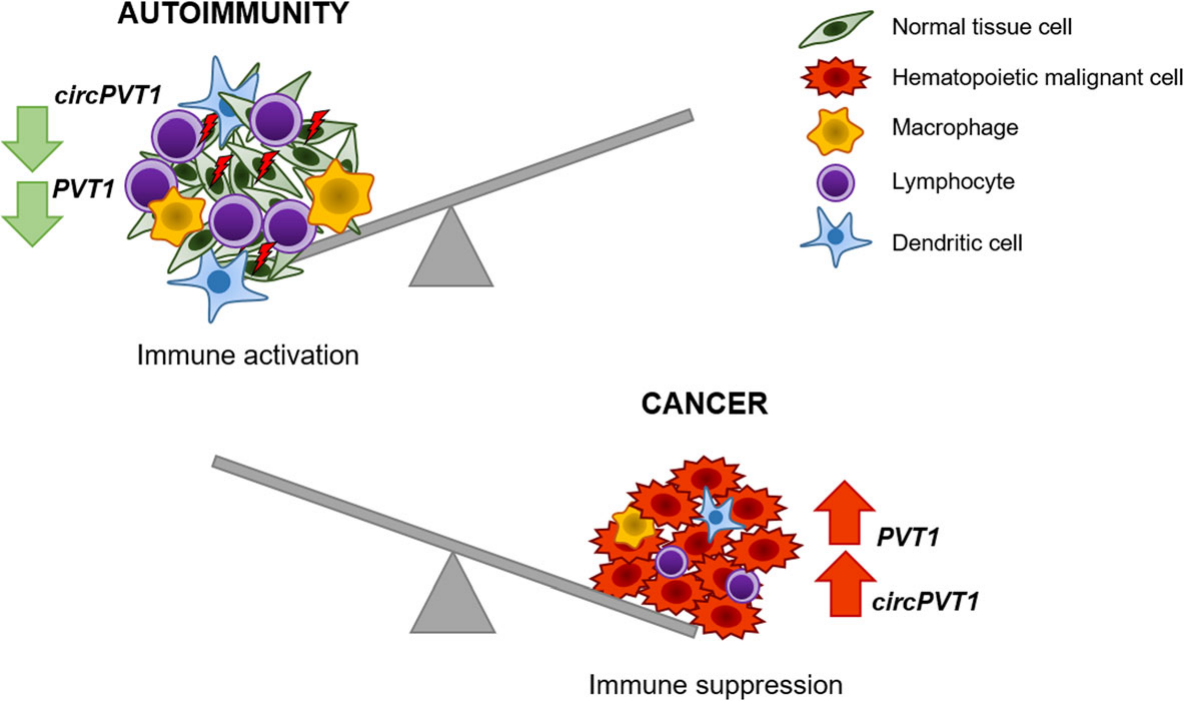
Consequences of *PVT1* and/or *circPVT1* deregulation in the hematopoietic system

The scenario depicted by recent data discussed in this review suggests a double edge of *PVT1* and *circPVT1* in the hematopoietic system: under non-tumorigenic conditions, *circPVT1* has a positive role in the regulation of protective immune responses and pathological effects in case of autoimmune reactions (Fig. 4). However, *PVT1* and *circPVT1* potentiate malignant cells, while hampering anti-tumor immune response during cancer progression (Fig. 4). No transforming ability of *PVT1* and *circPVT1* per se has been demonstrated in hematopoietic cells so far, suggesting a role in tumor progression and in support of a proliferative phenotype, rather than in cancer development. Moreover, some new insights on the role of *PVT1* in drug response are emerging, including the elevated expression of both *PVT1* and *circPVT1* in the vincristine-resistant AMU-ML2 DLBCL line [64] and the glucocorticoid-resistant phenotype promoted by *circPVT1* in MM models, that is rescued by knockdown [70]. Although some of these data need to be substantiated by scientific publications, they provide a clear path to go in hemato-oncology, where targeting of *PVT1* and/or *circPVT1* may a be a valuable option for combination therapies. Indeed, *PVT1*/*circPVT1*-mediated immune suppression is a challenge for those therapies aiming at restoring effective anti-tumor immune responses, including monoclonal antibodies (e.g.. αPD-1, αPD-L1, αCTLA-4) and cell-based therapies (e.g. chimeric antigen receptors [CAR]-T and CAR-cytokine induced killer cells) that are emerging as preferred candidates for combination therapies and the future of anti-cancer therapy, respectively.

Some key questions still need to be addressed regarding the role of *PVT1* and *circPVT1*. First, most studies did not account for the diverse *PVT1* isoforms and their differential expression across human cancers. Therefore, the most abundant variants expressed in the hematopoietic tissues may have been missed and little is known on the expression and function of *circPVT1* isoforms in the hematopoietic cells. Most functional studies show an intermediate phenotype, suggesting a potential compensatory role of secondary isoforms, which has not been elucidated yet. Second, similarities and differences between *PVT1* and the most studied *circPVT1* isoform remain vague. A similar role has been proposed in ALL and MM. However, their reciprocal regulation is still unexplored and the genomic localization of *circPVT1* does not facilitate the work. Their structural differences also suggest potentially diverse mechanisms of action, with *circPVT1* being a preferred candidate for extracellular localization (e.g. extracellular vesicles, cell-free RNA) and for the cross-talk between cancer cells and the immune system, that deserve future investigation. So far, 8/26 *PVT1* isoforms (although not including the ones expressed at high level in the hematopoietic tissues) have been detected in exosomes from cell lines, primary tumors and/or serum of patients with active tuberculosis (http://www.noncode.org), suggesting a functional role on the tumor microenvironment, especially under inflammatory conditions, that in turn favour tumor progression. Therefore, extracellular *PVT1* and especially *circPVT1* may have a putative role in hematological malignancies. In MM, extracellular vesicles have a supportive role during metastatization by promoting angiogenesis, uptake at distant premetastatic niches and activation of osteolytic activity [129]. Given their known role in angiogenesis, as proved in solid tumors, and their ability to shape cellular function, as demonstrated in immune cells, we can hypothesize that *PVT1* and *circPVT1* may be loaded as RNA cargo in extracellular vesicles released by plasma cells, in order to instruct the tumor microenvironment (e.g. fibroblasts) and promote bone metastasis.

A better understanding of all these topics will help to define targeted therapeutic interventions acting on the good and the bad of the hematopoietic system, with the aim of weakening the malignant cells and reactivate the anti-tumor immune response.

## Supplementary information

**Additional file 1: Table S1.** Core of *PVT1* coexpressed genes in adult and pediatric AML, pediatric ALL and DLBCL.

**Additional file 2: Table S2.** Pathway enrichment analysis of *PVT1* coexpressed genes.

## Data Availability

The datasets analysed during the current study are available in the [BioPortal for cancer genomics repository, [http://www.cbioportal.org/].

## Abbreviations

AEL: Acute erythroleukemia
ALL: Acute lymphoblastic leukemia
AML: Acute myeloid leukemia
AMPL-amp: AML cases carrying *MYC* amplifications as dmin, hsr or ring chromosomes
APL: Acute promyelocytic leukemia
ATRA: All-trans retinoic acid
BET: Bromodomain and extra-terminal domain
BL: Burkitt lymphoma
BM: Bone marrow
CAR: Chimeric antigen receptor
ceRNA: Competing endogenous RNA
circRNA: Circular RNA
CLL: Chronic lymphocytic leukemia
DLBCL: Diffuse large B cell lymphoma
dmin: Double minutes chromosomes
ds: Double stranded
FL: Follicular lymphoma
HL: Hodgkin’s lymphoma
hsr: Homogeneously staining region
Ig: Immunoglobulin
G-MDSC: Granulocytic myeloid-derived suppressor cells
IFN: Interferon
lncRNA: Long ncRNA
MLV: Murine leukemia virus
MM: Multiple myeloma
ncRNA: Non coding RNA
ORF: Open reading frame
PB: Peripheral blood
*PVT1*: Human *Plasmacytoma Variant Translocation 1*
ROS: Reactive oxygen species
SLE: Systemic lupus erythematosus
Treg: Regulatory T cells
