# Supplementary material for: Linear and circular PVT1 in hematological malignancies and immune response: two faces of the same coin
Source: Mol Cancer. 2020 Mar 30;19:69. doi: 10.1186/s12943-020-01187-5 (PMC7104523; doi:10.1186/s12943-020-01187-5)
Supplement: Supplementary file 1 — Additional file 1: Table S1. Core of PVT1 coexpressed genes in adult and pediatric AML, pediatric ALL and DLBCL. [file 12943_2020_1187_MOESM1_ESM.pdf]

Additional Table 1. Core of *PVT1* coexpressed genes in adult and pediatric AML, pediatric ALL and DLBCL.

Gene	Cytoband	Spearman's Correlation			
		adult AML	pediatric AML	pediatric ALL	DLBCL
MRPL37	1p32.3	0.317766	0.591173	0.406757	0.442358
CZIB	1p32.3	0.272893	0.546377	0.335849	0.389492
ERI3	1p34.1	0.222042	0.56693	0.401829	0.42716
MRPS15	1p34.3	0.238564	0.471542	0.301705	0.450065
AK2	1p35.1	0.457827	0.482082	0.315006	0.403278
ATP5IF1	1p35.3	0.260609	0.471805	0.400475	0.531806
MECR	1p35.3	0.215919	0.657444	0.40537	0.466131
ZNF593	1p36.11	0.347414	0.510013	0.327784	0.538102
MICOS10	1p36.13	0.375167	0.422925	0.296592	0.431394
AGMAT	1p36.21	0.308132	0.555863	0.264567	0.439861
PARK7	1p36.23	0.267451	0.522925	0.342812	0.439535
ATAD3A	1p36.33	0.26482	0.559816	0.395073	0.385258
AURKAIP1	1p36.33	0.231101	0.429776	0.322663	0.480243
MTX1	1q22	0.269077	0.518577	0.303818	0.457664
CCT3	1q22	0.225237	0.655863	0.380558	0.432696
MRPL24	1q23.1	0.272476	0.569038	0.282425	0.415653
JMJD4	1q42.13	0.240363	0.468643	0.334245	0.455601
ZNF692	1q44	0.258659	0.564427	0.312534	0.468411
IMMT	2p11.2|2	0.3797	0.436759	0.176539	0.396982
MDH1	2p15	0.23155	0.425296	0.281269	0.44366
COX5B	2q11.2	0.311606	0.4361	0.32024	0.416413
ATIC	2q35	0.237309	0.546904	0.484147	0.468085
PPP1R7	2q37.3	0.306036	0.451383	0.259907	0.408489
DTYMK	2q37.3	0.284592	0.585507	0.491746	0.396114
ACY1	3p21.2	0.423385	0.622661	0.348681	0.493595
RRP9	3p21.2	0.282916	0.662055	0.400454	0.420321
MON1A	3p21.31	0.300561	0.577339	0.386968	0.386018
APEH	3p21.31	0.276481	0.636627	0.367657	0.403713
UQCRC1	3p21.31	0.241924	0.579051	0.401644	0.444746
EEFSEC	3q21.3	0.240619	0.574177	0.370492	0.441706
PCCB	3q22.3	0.217193	0.539394	0.41331	0.47145
POLR2H	3q27.1	0.34882	0.624638	0.415233	0.450174
DANCR	4q12	0.238172	0.65942	0.280863	0.493378
NDUFA2	5q31.3	0.232381	0.497892	0.383392	0.468411
NOP16	5q35.2	0.363699	0.460211	0.369573	0.383739
NHP2	5q35.3	0.232767	0.603689	0.376679	0.452888
DNPH1	6p21.1	0.419333	0.63531	0.359108	0.512592
MRPL14	6p21.1	0.405488	0.491831	0.247685	0.390686
TOMM6	6p21.1	0.369123	0.555204	0.261835	0.513135
CCDC167	6p21.2	0.23731	0.433992	0.3368	0.541033
PFDN6	6p21.32	0.283669	0.571673	0.264743	0.448654
NELFE	6p21.33	0.233086	0.442029	0.367316	0.440187

NDUFAF4	6q16.1	0.26127	0.519104	0.306196	0.49924
HDDC2	6q22.31	0.354061	0.50224	0.390796	0.404798
PDCD2	6q27	0.219203	0.518972	0.355239	0.466782
MRPS24	7p13	0.276854	0.494335	0.267417	0.432805
POP7	7q22.1	0.323431	0.513834	0.441095	0.464937
AP1S1	7q22.1	0.286618	0.543347	0.267053	0.431611
MRPS28	8q21.13	0.347651	0.498287	0.260107	0.427703
NDUFB9	8q24.13	0.515007	0.579183	0.306472	0.412505
CYC1	8q24.3	0.376912	0.634387	0.363011	0.398936
TSTA3	8q24.3	0.372199	0.618577	0.352909	0.485128
SLC39A4	8q24.3	0.354491	0.658366	0.400558	0.498806
PUF60	8q24.3	0.340131	0.63834	0.355156	0.471559
SHARPIN	8q24.3	0.304778	0.625823	0.38706	0.544073
ADCK5	8q24.3	0.286504	0.600659	0.327512	0.432588
MFSD3	8q24.3	0.279155	0.628458	0.351578	0.531372
FBXL6	8q24.3	0.275008	0.626898	0.334307	0.500977
HSF1	8q24.3	0.239661	0.524901	0.265902	0.508142
HINT2	9p13.3	0.295364	0.560395	0.325224	0.428789
RPP25L	9p13.3	0.215593	0.526286	0.313	0.503582
DPM2	9q34.11	0.335737	0.507905	0.337644	0.422927
SH3GLB2	9q34.11	0.31055	0.498379	0.45214	0.394051
PHPT1	9q34.3	0.348545	0.596706	0.470733	0.426726
TPRN	9q34.3	0.329137	0.662579	0.372955	0.410334
MRPS2	9q34.3	0.24909	0.624506	0.331019	0.506839
SSNA1	9q34.3	0.226935	0.544186	0.384954	0.445506
TYSND1	10q22.1	0.240707	0.560606	0.278971	0.476552
COMTD1	10q22.2	0.334368	0.596838	0.255442	0.449739
CHCHD1	10q22.2	0.312582	0.427404	0.258537	0.522471
MRPS16	10q22.2	0.237139	0.552042	0.381476	0.393074
NDUFB8	10q24.31	0.380987	0.544796	0.264484	0.469713
NPM3	10q24.32	0.289049	0.561792	0.271702	0.46244
DPCD	10q24.32	0.261374	0.435178	0.242417	0.404147
SFXN4	10q26.11	0.308577	0.556258	0.430554	0.432045
ECHS1	10q26.3	0.342843	0.571278	0.371104	0.580004
PSMC3	11p11.2	0.220999	0.50975	0.387061	0.404907
POLR2L	11p15.5	0.287313	0.453623	0.326086	0.386235
MRPL23	11p15.5	0.254404	0.46917	0.31424	0.508033
UQCC3	11q12.3	0.241874	0.624506	0.380419	0.430634
TMEM223	11q12.3	0.225181	0.472596	0.237541	0.449739
CCDC85B	11q13.1	0.25155	0.565876	0.349141	0.544616
RNASEH2C	11q13.1	0.240398	0.590777	0.368887	0.414785
MRPL11	11q13.2	0.253399	0.516996	0.389967	0.45799
MRPL51	12p13.31	0.236383	0.502503	0.37615	0.429766
TSFM	12q14.1	0.239746	0.422134	0.293137	0.384064
MRPL52	14q11.2	0.21705	0.424506	0.351543	0.447026
TRMT61A	14q32.33	0.237742	0.617787	0.31124	0.40013
WDR61	15q25.1	0.264889	0.586166	0.375819	0.454624
TUFM	16p11.2	0.235384	0.597233	0.332821	0.433891
MRPS34	16p13.3	0.329522	0.622661	0.317938	0.491533

STUB1	16p13.3	0.294255	0.513966	0.320211	0.416413
MLST8	16p13.3	0.268948	0.617655	0.41155	0.482414
METTL26	16p13.3	0.259275	0.645586	0.364536	0.51498
ECI1	16p13.3	0.223584	0.594335	0.400951	0.45495
COQ9	16q21	0.227817	0.557708	0.33611	0.392531
CTU2	16q24.3	0.341935	0.489196	0.264111	0.420864
TRAPPC2L	16q24.3	0.300691	0.442424	0.306922	0.403061
APRT	16q24.3	0.244697	0.567589	0.325802	0.441164
TMEM256	17p13.1	0.282316	0.42108	0.322908	0.537234
NT5C3B	17q21.2	0.286883	0.422793	0.293211	0.428029
ATP5MC1	17q21.32	0.314413	0.62253	0.373281	0.516283
NME1	17q21.33	0.331226	0.680501	0.375137	0.421515
NME2	17q21.33	0.275102	0.44809	0.225281	0.481871
MRPL27	17q21.33	0.253595	0.522661	0.309031	0.452453
FDXR	17q25.1	0.431999	0.741634	0.42596	0.428137
MRPL58	17q25.1	0.294011	0.573518	0.288289	0.433673
ATP5PD	17q25.1	0.243919	0.510804	0.298635	0.433131
MRPL12	17q25.3	0.357178	0.697365	0.401763	0.521602
NDUFAF8	17q25.3	0.322701	0.622266	0.368819	0.537017
RFNG	17q25.3	0.257312	0.428854	0.23968	0.528224
ANAPC11	17q25.3	0.248098	0.451515	0.311464	0.40469
DUS1L	17q25.3	0.247842	0.523452	0.17218	0.444963
MRPL34	19p13.11	0.319502	0.45863	0.315139	0.476878
TMEM161A	19p13.11	0.311546	0.587615	0.365224	0.420864
LSM4	19p13.11	0.272074	0.431225	0.352247	0.453322
CCDC124	19p13.11	0.262654	0.618709	0.308732	0.484151
NDUFB7	19p13.12	0.271581	0.445586	0.365997	0.421624
GADD45GIP1	19p13.13	0.237268	0.572464	0.382539	0.484477
YIPF2	19p13.2	0.290044	0.429908	0.266905	0.447243
ECSIT	19p13.2	0.286406	0.599078	0.4352	0.416196
MRPL4	19p13.2	0.270686	0.621871	0.38647	0.459509
QTRT1	19p13.2	0.244836	0.599868	0.341378	0.544833
MICOS13	19p13.3	0.326529	0.564822	0.30813	0.467108
PLEKHJ1	19p13.3	0.293841	0.589592	0.311987	0.512918
MAP2K2	19p13.3	0.259029	0.419895	0.34049	0.514003
TIMM13	19p13.3	0.256566	0.557839	0.292637	0.501086
NDUFA11	19p13.3	0.246217	0.486561	0.371548	0.438233
POLR2E	19p13.3	0.213902	0.536759	0.356278	0.422058
PDCD2L	19q13.11	0.309577	0.533333	0.375644	0.394377
MRPS12	19q13.2	0.334144	0.620685	0.322507	0.461246
ECH1	19q13.2	0.267257	0.527273	0.340404	0.412288
ERCC2	19q13.32	0.406259	0.595652	0.349343	0.417933
BCAT2	19q13.33	0.235998	0.559025	0.427493	0.442358
ISOC2	19q13.42	0.339631	0.529249	0.437052	0.417173
HSPBP1	19q13.42	0.232059	0.478524	0.395321	0.439861
ITPA	20p13	0.323956	0.520817	0.345036	0.465263
MRPS26	20p13	0.250036	0.62029	0.341971	0.447351
NECAB3	20q11.22	0.333059	0.470487	0.178348	0.459835
EIF6	20q11.22	0.27722	0.553623	0.291983	0.416848

FAM207A	21q22.3	0.259964	0.605929	0.247962	0.477421
RRP1	21q22.3	0.257442	0.575362	0.216439	0.51726
MRPL40	22q11.21	0.28536	0.563505	0.34129	0.427812
TXNRD2	22q11.21	0.229122	0.424769	0.230943	0.561876
RANBP1	22q11.21	0.21003	0.562055	0.43521	0.400564
MIF	22q11.23	0.230839	0.50224	0.328572	0.539731
TXN2	22q12.3	0.283637	0.595125	0.38237	0.500326
ZBTB18	1q44	-0.30258	-0.47918	-0.23537	-0.41066
ITPRID2	2q31.3	-0.25407	-0.44862	-0.26053	-0.43888
TMF1	3p14.1	-0.29412	-0.42069	-0.3388	-0.40447
XRN1	3q23	-0.22674	-0.49407	-0.2692	-0.46863
PIK3CA	3q26.32	-0.24712	-0.5054	-0.33902	-0.46591
TLR1	4p14	-0.25292	-0.52885	-0.30492	-0.39231
CNOT6L	4q21.1	-0.42435	-0.5224	-0.40224	-0.44974
DNAJB14	4q23	-0.21048	-0.43597	-0.31453	-0.4505
PJA2	5q21.3	-0.23907	-0.49051	-0.35629	-0.4099
CDC42SE2	5q31.1	-0.275	-0.53505	-0.23854	-0.47264
RC3H2	9q33.2	-0.25405	-0.44348	-0.2328	-0.46635
CCSER2	10q23.1	-0.29253	-0.53149	-0.22962	-0.40339
TNKS2	10q23.32	-0.24349	-0.47207	-0.33377	-0.45289
UBL3	13q12.3	-0.22757	-0.4776	-0.3895	-0.39948
SECISBP2L	15q21.1	-0.33806	-0.46179	-0.30031	-0.49577
PIAS1	15q23	-0.24941	-0.43426	-0.28275	-0.46494
IREB2	15q25.1	-0.30425	-0.47958	-0.2932	-0.47188
AKAP13	15q25.3	-0.21147	-0.43794	-0.28129	-0.46743
CYLD	16q12.1	-0.21333	-0.51673	-0.42752	-0.39058
SMURF2	17q23.3-q24.1	-0.27926	-0.54585	-0.37692	-0.5432
PPP1R16B	20q11.23	-0.23771	-0.56192	-0.18301	-0.41251
P2RY10	Xq21.1	-0.22269	-0.5401	-0.2556	-0.415
