# Supplementary material for: Linear and circular PVT1 in hematological malignancies and immune response: two faces of the same coin
Source: Mol Cancer. 2020 Mar 30;19:69. doi: 10.1186/s12943-020-01187-5 (PMC7104523; doi:10.1186/s12943-020-01187-5)
Supplement: Supplementary file 2 — Additional file 2: Table S2. Pathway enrichment analysis of PVT1 coexpressed genes. [file 12943_2020_1187_MOESM2_ESM.pdf]

Additional Table 2. Pathway enrichment analysis of *PVT1* coexpressed genes.

Database	Term	Adjusted P-value	Combined Score	Genes
**Transcription and RNA metabolism**				
GO-BP	transcription from RNA polymerase I promoter	0.038437	117.49	ERCC2;POLR2E;POLR2H;POLR2L
Reactome	RNA Pol II CTD phosphorylation and interaction with CE	0.005657	163.97	ERCC2;POLR2E;POLR2H;POLR2L
Reactome	RNA Pol II CTD phosphorylation and interaction with CE	0.0054	163.97	ERCC2;POLR2E;POLR2H;POLR2L
Reactome	mRNA Capping	0.006898	148.00	ERCC2;POLR2E;POLR2H;POLR2L
Reactome	RNA Polymerase I Promoter Escape	0.007577	140.94	ERCC2;POLR2E;POLR2H;POLR2L
Reactome	RNA Polymerase II Pre-transcription Events	0.01045	77.01	ERCC2;POLR2E;POLR2H;NELFE;POLR2L
Reactome	RNA Polymerase II Transcription Pre-Initiation And Promoter Opening	0.020017	80.07	ERCC2;POLR2E;POLR2H;POLR2L
Reactome	RNA Polymerase II Transcription Initiation And Promoter Clearance	0.021043	80.07	ERCC2;POLR2E;POLR2H;POLR2L
Reactome	RNA Polymerase II Transcription Initiation	0.020517	80.07	ERCC2;POLR2E;POLR2H;POLR2L
GO-BP	transcription initiation from RNA polymerase I promoter	0.037013	117.49	ERCC2;POLR2E;POLR2H;POLR2L
Reactome	RNA Polymerase III Transcription Initiation From Type 1 Promoter	0.045352	79.77	POLR2E;POLR2H;POLR2L
Reactome	RNA Polymerase III Transcription Initiation From Type 2 Promoter	0.042203	84.12	POLR2E;POLR2H;POLR2L
Reactome	RNA Polymerase III Transcription Initiation From Type 3 Promoter	0.044583	79.77	POLR2E;POLR2H;POLR2L
Reactome	Formation of the Early Elongation Complex	0.001289	207.06	ERCC2;POLR2E;POLR2H;NELFE;POLR2L
Reactome	Formation of RNA Pol II elongation complex	0.003037	136.09	ERCC2;POLR2E;POLR2H;NELFE;POLR2L
Reactome	RNA Polymerase I Chain Elongation	0.040573	53.75	ERCC2;POLR2E;POLR2H;POLR2L
Reactome	RNA Polymerase II Transcription Elongation	0.003216	136.09	ERCC2;POLR2E;POLR2H;NELFE;POLR2L
Reactome	RNA Polymerase III Chain Elongation	0.019932	149.96	POLR2E;POLR2H;POLR2L
GO-BP	transcription elongation from RNA polymerase I promoter	0.026457	148.00	ERCC2;POLR2E;POLR2H;POLR2L

GO-BP	transcription elongation from RNA polymerase II promoter	0.035851	63.21	ERCC2;POLR2E;WDR61;POLR2H;NELFE;POLR2L
Reactome	RNA Polymerase I Transcription Termination	0.00872	128.35	ERCC2;POLR2E;POLR2H;POLR2L
Reactome	RNA Polymerase III Transcription Termination	0.030568	106.02	POLR2E;POLR2H;POLR2L
Reactome	Elongation arrest and recovery	0.007848	128.35	POLR2E;POLR2H;NELFE;POLR2L
Reactome	Deadenylation-dependent mRNA decay	0.028467	65.89	NT5C3B;CNOT6L;XRN1;LSM4
GO-BP	7-methylguanosine mRNA capping	0.038595	122.73	ERCC2;POLR2E;POLR2H;POLR2L
GO-BP	7-methylguanosine RNA capping	0.036986	122.73	ERCC2;POLR2E;POLR2H;POLR2L
Reactome	Formation of TC-NER Pre-Incision Complex	0.033355	60.21	ERCC2;POLR2E;POLR2H;POLR2L
Reactome	Gap-filling DNA repair synthesis and ligation in TC-NER	0.049074	47.06	ERCC2;POLR2E;POLR2H;POLR2L
Reactome	Formation of TC-NER Pre-Incision Complex	0.033355	60.21	ERCC2;POLR2E;POLR2H;POLR2L
Reactome	Gap-filling DNA repair synthesis and ligation in TC-NER	0.049074	47.06	ERCC2;POLR2E;POLR2H;POLR2L
**Translation**				
KEGG	Ribosome	2.48E-06	170.97	MRPL4;MRPS15;MRPS16;MRPS12;MRPL27;MRPS2;MRPL14;MRPL12;MRPL23;MRPL34;MRPL24;MRPL11
GO-BP	translation	9.93E-12	322.08	EEFSEC;TSFM;MRPS15;MRPS16;MRPS24;MRPS12;MRPS34;MRPL27;MRPS2;MRPL37;APEH;MRPL23;MRPL34;MRPL24;TUFM;MRPL11;MRPL4;MRPL52;MRPL51;NHP2
GO-BP	translational elongation	2.92E-29	2191.06	MRPS15;MRPS16;MRPS12;MRPS34;MRPL14;MRPL58;MRPL37;MRPL12;MRPL34;MRPL11;MRPL4;MRPL40;CHCHD1;EEFSEC;MRPS28;GADD45GIP1;TSFM;MRPS26;MRPS24;MRPL27;MRPS2;MRPL23;MRPL24;TUFM;MRPL52;MRPL51;AURKAIP1
GO-BP	translational termination	2.48E-27	2062.28	MRPS15;MRPS16;MRPS12;MRPS34;MRPL14;MRPL58;MRPL37;APEH;MRPL12;MRPL34;MRPL11;MRPL4;MRPL40;CHCHD1;MRPS28;GADD45GIP1;MRPS26;MRPS24;MRPL27;MRPS2;MRPL23;MRPL24;MRPL52;MRPL51;AURKAIP1
Reactome	Mitochondrial translation	9.13E-27	2077.14	MRPS28;GADD45GIP1;TSFM;MRPS15;MRPS26;MRPS16;MRPS24;MRPS12;MRPS34;MRPL27;MRPL14;MRPL37;MRPL34;MRPL12;MRPL23;MRPL24;TUFM;MRPL11;MRPL4;MRPL52;MRPL40;MRPL51;AURKAIP1;CHCHD1
GO-BP	mitochondrial translation	1.54E-27	1947.82	MRPS15;MRPS16;MRPS12;MRPS34;MRPL14;MRPL58;MRPL37;MRPL12;MRPL34;MRPL11;MRPL4;MRPL40;CHCHD1;MRPS28;GADD45GIP1;TSFM;MRPS26;MRPS24;MRPL27;M

				RPS2;MRPL23;MRPL24;TUFM;MRPL52;MRPL51;AURKAIP1
Reactome	Mitochondrial translation initiation	2.40E-24	1856.90	MRPS28;GADD45GIP1;MRPS15;MRPS26;MRPS16;MRPS24;MRPS12;MRPS34;MRPL27;MRPL14;MRPL37;MRPL34;MRPL12;MRPL23;MRPL24;MRPL11;MRPL4;MRPL52;MRPL40;MRPL51;AURKAIP1;CHCHD1
Reactome	Mitochondrial translation elongation	2.78E-27	2288.40	MRPS28;GADD45GIP1;TSFM;MRPS15;MRPS26;MRPS16;MRPS24;MRPS12;MRPS34;MRPL27;MRPL14;MRPL37;MRPL34;MRPL12;MRPL23;MRPL24;TUFM;MRPL11;MRPL4;MRPL52;MRPL40;MRPL51;AURKAIP1;CHCHD1
GO-BP	mitochondrial translational elongation	1.12E-29	2598.47	MRPS15;MRPS16;MRPS12;MRPS34;MRPL14;MRPL58;MRPL37;MRPL34;MRPL12;MRPL11;MRPL4;MRPL40;CHCHD1;MRPS28;GADD45GIP1;TSFM;MRPS26;MRPS24;MRPL27;MRPS2;MRPL23;MRPL24;TUFM;MRPL52;MRPL51;AURKAIP1
Reactome	Mitochondrial translation termination	1.80E-24	1856.90	MRPS28;GADD45GIP1;MRPS15;MRPS26;MRPS16;MRPS24;MRPS12;MRPS34;MRPL27;MRPL14;MRPL37;MRPL34;MRPL12;MRPL23;MRPL24;MRPL11;MRPL4;MRPL52;MRPL40;MRPL51;AURKAIP1;CHCHD1
GO-BP	mitochondrial translational termination	1.22E-26	2077.14	MRPS28;GADD45GIP1;MRPS15;MRPS26;MRPS16;MRPS24;MRPS12;MRPS34;MRPL27;MRPS2;MRPL14;MRPL58;MRPL37;MRPL34;MRPL12;MRPL23;MRPL24;MRPL11;MRPL4;MRPL52;MRPL40;MRPL51;AURKAIP1;CHCHD1
**Metabolism**				
Reactome	Metabolism of nucleotides	0.001238	108.62	DTYMK;ATIC;NME2;AK2;ITPA;NME1;APRT
KEGG	Purine metabolism	0.004737	57.14	NT5C3B;ATIC;AK2;NME2;ITPA;NME1;APRT
Hu Cyc 2016	superpathway of purine nucleotide salvage	0.017779	77.39	AK2;NME2;APRT;NME1
Hu Cyc 2016	purine nucleotides de novo biosynthesis	0.014816	77.39	ATIC;AK2;NME2;NME1
KEGG	Pyrimidine metabolism	0.049006	53.75	NT5C3B;DTYMK;NME2;NME1
Hu Cyc 2016	pyrimidine deoxyribonucleotide phosphorylation	0.014645	294.98	DTYMK;NME2;NME1
Hu Cyc 2016	pyrimidine deoxyribonucleotides biosynthesis from CTP	0.01969	193.18	DTYMK;NME2;NME1
Hu Cyc 2016	pyrimidine deoxyribonucleotides de novo biosynthesis	0.013127	193.18	DTYMK;NME2;NME1
Hu Cyc 2016	superpathway of pyrimidine deoxyribonucleoside salvage	0.012041	176.69	DTYMK;NME2;NME1

Hu Cyc 2016	superpathway of pyrimidine deoxyribonucleotides de novo biosynthesis	0.015836	120.69	DTYMK;NME2;NME1
KEGG	Oxidative phosphorylation	1.69E-05	134.85	NDUFB9;NDUFB8;NDUFB7;ATP5PD;NDUFA11;NDUFA2;UQCRC1;CYC1;COX5B;ATP5MC1
Reactome	The citric acid (TCA) cycle and respiratory electron transport	1.37E-04	107.28	NDUFB9;NDUFB8;NDUFB7;NDUFA11;NDUFAF4;NDUFA2;UQCRC1;CYC1;ECSIT;COX5B
Reactome	Respiratory electron transport	1.04E-06	256.77	NDUFB9;NDUFB8;NDUFB7;NDUFA11;NDUFAF4;NDUFA2;UQCRC1;CYC1;ECSIT;COX5B
Reactome	Respiratory electron transport, ATP synthesis by chemiosmotic coupling, and heat production by uncoupling proteins.	7.21E-06	184.85	NDUFB9;NDUFB8;NDUFB7;NDUFA11;NDUFAF4;NDUFA2;UQCRC1;CYC1;ECSIT;COX5B
Reactome	Complex I biogenesis	3.59E-05	258.81	NDUFB9;NDUFB8;NDUFB7;NDUFA11;NDUFAF4;NDUFA2;ECSIT
GO-BP	mitochondrial ATP synthesis coupled electron transport	2.40E-05	205.36	NDUFB9;NDUFB8;NDUFB7;NDUFA11;UQCC3;NDUFA2;UQCRC1;CYC1;COX5B
GO-BP	NADH dehydrogenase complex assembly	3.44E-05	235.09	NDUFB9;NDUFB8;NDUFAF8;NDUFB7;NDUFA11;NDUFAF4;NDUFA2;ECSIT
GO-BP	mitochondrial respiratory chain complex I biogenesis	3.17E-05	235.09	NDUFB9;NDUFB8;NDUFAF8;NDUFB7;NDUFA11;NDUFAF4;NDUFA2;ECSIT
GO-BP	mitochondrial respiratory chain complex I assembly	2.94E-05	235.09	NDUFB9;NDUFB8;NDUFAF8;NDUFB7;NDUFA11;NDUFAF4;NDUFA2;ECSIT
GO-BP	respiratory electron transport chain	4.21E-05	176.24	NDUFB9;NDUFB8;NDUFB7;NDUFA11;UQCC3;NDUFA2;UQCRC1;CYC1;COX5B
GO-BP	mitochondrial respiratory chain complex assembly	5.17E-05	167.94	NDUFB9;NDUFAF8;NDUFB8;NDUFB7;NDUFA11;UQCC3;NDUFAF4;NDUFA2;ECSIT
GO-BP	mitochondrial electron transport, NADH to ubiquinone	0.013276	123.37	NDUFB9;NDUFB8;NDUFB7;NDUFA11;NDUFA2
GO-BP	mitochondrial electron transport, ubiquinol to cytochrome c	0.038012	212.41	UQCC3;UQCRC1;CYC1
Hu Cyc 2016	isoleucine degradation	0.049226	105.19	ECHS1;BCAT2
Reactome	Mitochondrial Fatty Acid Beta-Oxidation	0.017206	162.42	ECHS1;ECI1;PCCB
